# Expression profiles of the *MXD3* gene and association of sequence variants with growth traits in Xianan and Qinchuan cattle

**DOI:** 10.1002/vms3.251

**Published:** 2020-03-05

**Authors:** Dan Hao, Bo Thomsen, Jiangsong Bai, Shujun Peng, Xianyong Lan, Yongzhen Huang, Xiao Wang, Hong Chen

**Affiliations:** ^1^ College of Animal Science and Technology Northwest A&F University Shaanxi Key Laboratory of Animal Genetics, Breeding and Reproduction Yangling Shaanxi China; ^2^ Department of Molecular Biology and Genetics Aarhus University Aarhus C Denmark; ^3^ College of Veterinary Medicine China Agricultural University Beijing China; ^4^ Beijing Zhongnongtongchuang (ZNTC) Biotechnology Co., Ltd Beijing China; ^5^ Department of Applied Mathematics and Computer Science Technical University of Denmark Kongens Lyngby Denmark

**Keywords:** cattle, expression level, growth trait, *MXD3* gene

## Abstract

Max dimerization protein 3 (MXD3) belongs to the MYC superfamily of basic helix‐loop‐helix leucine zipper transcription factors, and MXD3‐MAX heterodimers can bind to promoters of target genes to modulate their expression. The aim of this study was to determine the *MXD3* mRNA expression levels in various cattle tissues comprising heart, liver, spleen, lung, kidney, *Longissimus dorsi* muscle and subcutaneous fat in Chinese Qinchuan and Xianan cattle breeds. The RT‐qPCR data showed that the *MXD3* gene was variably expressed between all tissues and at levels that were significantly different between two breeds (*p* < .05). We used the polymerase chain reaction‐restriction fragment length polymorphism (PCR‐RFLP) method to investigate the possible association between single‐nucleotide polymorphisms (SNP) within the *MXD3* gene and five different growth traits in cattle. We found two intronic SNPs (g.2694 C>T and g.3801 T>C) and one SNP in 3′untranslated region (3′UTR) (g.6263 G>A) of *MXD3* gene. Association analysis revealed strong associations between pairwise and triple SNP combinations and the growth traits. Based on these results, we suggest that *MXD3* polymorphisms could be useful as molecular markers in the Chinese beef cattle breeding program.

## Graphical abstract

Three single‐nucleotide polymorphisms in *MXD3* and its expression level in various tissues of cattle.

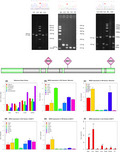

## 1 INTRODUCTION

The application of molecular genetics in animal husbandry has many important advantages (Khodabakhshzadeh et al., 2016; Zamani, Akhondi, & Mohammadabadi, 2015). One such significant advantage is the genotyping of individuals for specific genetic loci known to be associated with phenotypic traits of relevance to cattle farming and breeding (Khodabakhshzadeh et al., 2016; Mohammadreza Esfandyarpoo, & Mousapour, 2017). Often all the genes that affect a polygenic trait are not precisely known, although a number of candidate genes with major effects have been recognized (Mohammadabadi et al., 2010). In the candidate gene approach, the process of identifying such genes responsible for a polygenic trait variation includes the selection of candidate genes based on the relationship between physiological or biochemical processes involved in the expression of the phenotype, and subsequent testing of the selected genes as putative quantitative trait loci (QTL) (Mousavizadeh et al., 2009; Ruzina et al., 2010). The bovine genome is densely covered by single‐nucleotide polymorphisms (SNP) markers, which facilitates the search for genes with significant effects on quantitative trait variation (Javanmard et al., 2008). Integrated approaches in terms of management and genetic improvement are of crucial importance for enhancement of production (Mohammadabadi & Sattayimokhtari, 2013; Soufy et al., 2009). Furthermore, economical and biological efficiency of production enterprises generally improves by increasing productivity and reproductive performance of animals (Mohammadabadi & Sattayimokhtari, 2013; Taghi Vajed Ebrahimi, Mohammadabadi, & Esmailizadeh, 2017; Zamani et al., 2015). Growth phenotypes such as body weight and size of cattle are often used as selection criteria because of their association with meat production (Buzanskas et al., 2014). Complex traits are usually highly polygenic; a recent meta‐analysis for cattle stature identified 163 genome‐wide significant loci (Bouwman et al., 2018). Cattle selection programmes based on the molecular genetic information is a powerful and effective strategy to enhance economic quantitative traits. Thus, marker‐assisted selection (MAS) at the DNA level significantly increases selection accuracy of purposeful phenotypes and shortens the generation intervals (Bouquet & Juga, 2013). As one of the largest beef producer in the world, China began directional selection in beef cattle for melioration of meat performance after 1980s to satisfy an increasing consumption of beef (Waldron, Jimin, Huijie, Xiaoxia, & Tre, 2013). However, Chinese beef production still relies on imports from abroad as well as on collaborations between breeders to improve Chinese beef production (Waldron et al., 2013). The need to maintain and improve local genetic resources has been recognized as a priority, at the world level. For example, biodiversity studies depicting a deep picture of the genetic variability of the available sheep breeds provide favourable opportunities for both genetic conservation programmes as well as for enhancing production efficiency by means of controlled and well‐designed crossbreeding systems exploiting breed diversities, heterosis and breed complementarity (Taghi Vajed Ebrahimi et al., 2017). Genetic diversity in indigenous breeds is a major concern considering the necessity of preserving what may be a precious and irreplaceable richness, regarding future productive demands (Khodabakhshzadeh et al., 2016). Conservation should be based on a deep knowledge of the genetic resources of the specific breed (Mohammadreza et al., 2017; Zamani et al., 2015). Therefore, it is important to characterize genetically indigenous breeds. Genes affecting polygenic traits and characterizing milk or meat production are difficult to identify (Shojaei et al., 2011; Soufy et al., 2009). The maintenance of genetic diversity in livestock species requires the adequate implementation of conservation priorities and sustainable management programmes, which should be based on comprehensive information regarding the structure of the populations, including sources of genetic variability among and within breeds (Mousavizadeh et al., 2009; Ruzina et al., 2010). Genetic diversity is an essential component for population survival, evolution, genetic improvement and adaptation to changing environmental conditions (Taghi Vajed Ebrahimi et al., 2017), and molecular methods based on molecular markers, such as RAPD, RFLP and microsatellites, are useful tools to study the underlying genetics (Mohammadabadi et al., 2010; Mousavizadeh et al., 2009; Taghi Vajed Ebrahimi et al., 2017).

The basic helix‐loop‐helix leucine zipper transcriptional regulators, which belongs to the MYC‐MAX‐MAD network, are central players in the control of cell‐cycle progression, proliferation, apoptosis and transformation (Grandori, Cowley, James, & Eisenman, 2000). The MAX‐MYC heterodimer is a sequence‐specific transcriptional activator, whereas the MAX‐MAD complex acts as a sequence‐specific transcriptional repressor (Lüscher & Vervoorts, 2012). The Max dimerization protein 3 (MXD3), member of MAD family, has been considered a potential target for therapeutic treatment of brain and central nervous system cancers due to its role in cellular proliferation and tumorigenesis (Barisone et al., 2012). *MXD3* expression is significantly upregulated in visceral adipose tissues in human obese adults as well as in a zebrafish model of diet‐induced obesity in which downregulation of *MXD3* expression suppressed the formation of visceral adiposity (Shimada et al., 2014). In addition, the expression of *MXD3* was three‐fold reduced in adult skeletal muscle tissues compared with the fetal period in Qinchuan (QC) cattle. *MXD3* gene was also enriched in the Gene Ontology (GO) terms of DNA binding, protein binding, negative regulation of transcription, DNA‐dependent and protein dimerization activity (Li et al., 2017). Therefore, the objective of this study was to identify SNPs within *MXD3* and then to analyze their associations with the growth traits of Chinese cattle. We studied two different breeds, the QC breed, which is one of the most important Chinese beef cattle breeds, yet exhibiting worse growth performance and carcass traits than imported European cattle breeds (Xie, Meng, Cui, & Ren, 2012; Xie, Meng, Ren, Shi, & Zhou, 2012), as well as the Xianan (XN) cattle which is a crossbreed between Charolais cattle and Nanyang cattle.

To the best our knowledge, our study provided the first association analysis of *MXD3* sequence variations with growth traits and expression profiles of *MXD3* in seven tissues under the best normalized reference genes in cattle, which may improve the understanding of the molecular basis and the application of MAS for beef cattle breeding in China.

## 2 MATERIALS AND METHODS

### 2.1 Cattle and data collection

A total of 499 samples of QC cattle (2‐year‐old, *n* = 141; 3.5‐year‐old, *n* = 181) and XN cattle (*n* = 177, 2‐year‐old) were collected from two farms: the farm of Fufeng country in Shaanxi and the farm of Nanyang city in Henan provinces of China, respectively. All of the individuals were females. Five growth traits for association analysis were recorded at the age of 2 years old, females with no pregnancy both in QC (*n* = 141) and XN (*n* = 170) cattle following Gilbert's method (Gilbert, Bailey, & Shannon, 1993).

The growth traits were body weight (BW, kg), body length (BL, cm), body height (BH, cm), chest circumference (ChC, cm) and hip cross height (HCH, cm). The genomic DNA from each cattle was purified from 2% heparin‐treated jugular blood samples and diluted to 50 ng/µl and subsequently stored at −20°C for further usage following the standard procedures (Sambrook & Russell 2001).

### 2.2 Detection and identification of the variants within *MXD3* gene

Using the bovine genome sequences in GenBank (Accession No. AC_000164), seven pairs of primer were designed by Primer v5.0 software (PREMIER Biosoft International) (Table S1). Genomic DNA pools of 40 randomly selected individuals were mixed gently from the two breeds, amplified and sequenced to identify the polymorphisms within *MXD3*. After comparing the *MXD3* sequence to National Center for Biotechnology Information (NCBI), three mutations within bovine *MXD3* were found. Accordingly, pairs of primers were designed to genotype the mutations based on restriction fragment length polymorphism (PCR‐RFLP) method using restriction enzyme *Taq* I, *Hha* I and *Pvu* II (Thermo Fisher Scientific) (Table S2).

### 2.3 RNA isolation and qRT‐PCR analysis

Seven tissues (heart, liver, spleen, lung, kidney, *longissimus dorsi* muscle and subcutaneous fat) were collected from three QC and three XN cattle when they were slaughtered at the cattle plant. After washing with phosphate buffered saline (PBS), samples were put immediately into liquid nitrogen and stored at −70°C for the subsequent steps; each tissues had three replicates. Next, Trizol reagent (Takara Co., Ltd) was used to extract the RNA. The RNA purity and concentration were determined using a NanoDrop 2000 spectrophotometer and we randomly chose three samples to check the purified RNA by 0.8% agarose gel electrophoresis (Figure S1). Next, the StarScript II One‐step RT‐PCR Kit (Takara Co., Ltd) with 2 µg RNA as the template was used for cDNA synthesis. Accurate normalization is a prerequisite for analysing target gene expression under various experimental conditions and samples. The RefFinder software (https://www.heartcure.com.au/reffinder/?type=reference) (Xie, Xiao, Chen, Xu, & Zhang, 2012) can conveniently and efficiently assess four software applications termed geNorm (Excel‐based) (Vandesompele et al., 2002), BestKeeper (Excel‐based) (Pfaffl, Tichopad, Prgomet, & Neuvians, 2004) and NormFinder (Excel‐based) (Andersen, Jensen, & Ørntoft, 2004). The standardization algorithms termed deltaCt method (Silver, Best, Jiang, & Thein, 2006) was also assessed in RefFinder. Herein, we employed RefFinder to assess the numbers and expression stability of reference genes. *GAPDH* (glyceraldehyde‐3‐phosphate dehydrogenase), *ACTB* (beta actin), *RPL19* (ribosomal protein 19) and *EMD* (emerin) were selected as the most appropriate reference genes and the relevant primers used in qRT‐PCR were shown in Table S1. The melt curve was added automatically to verify amplification efficiency and no template control reactions (Bio‐Rad). The 0.005, 0.05, 0.5, 5 and 50 ng cDNA were used to test the amplification efficiency. The correlation coefficient (*r* ^2^) was between 0.9312 and 0.9992, whereas the slope was −1.922 to −2.925 (Figure S2). The gene expression levels were calculated based on geNorm and 2^−ΔΔ^ *^C^* ^T^ method and we performed two‐tailed Student's *t* test to compare the differential expressions in various tissues from two adult cattle breeds.

### 2.4 Statistical analyses

Hardy–Weinberg equilibrium (HWE) was tested using the Predictive Analytics Software (PASW) (version 18) based on the chi‐squared test. Population genetic coefficients including effective allele numbers (*Ae*), expected heterozygosis (*He*) and polymorphism information content (*PIC*) were analysed based on Nei's methods (Nei & Roychoudhury, 1974). Linkage disequilibrium (LD) and haplotypes were analysed by SHEsis software (http://analysis.bio‐x.cn) (Li et al., 2009). We used 499 samples, including 322 QC and 177 XN cattle for diversity analyses, LD and haplotypes. Association analysis was evaluated using linear model by R package *multcomp* (version 1.4‐10) in 311 samples including QC (*n* = 141) and XN (*n* = 170) cattle at the same age (2 year‐old). The preliminary statistical analyses indicated that birth years, seasons and farms did not have significant associations with growth traits, so we used two‐way analysis of variance (ANOVA) for the five growth traits in 2‐year‐old QC and XN cattle. The models were as follows:

$$Y_{\mathrm{ij}}=\mu +{\text{Breed}}_{i}+{\text{Marker}}_{j}+e_{\mathrm{ij}}$$

where *Y* is the growth trait, *μ* is the overall mean of each trait, Breed is the cattle breed (i.e. QC and XN cattle), Marker is the SNP (SNP1, SNP2 and SNP3) and the pairwise interacted SNP (SNP1‐SNP2, SNP1‐SNP3, SNP2‐SNP3 and SNP1‐SNP2‐SNP3) and *e* is the residual error. Multiple comparisons were performed for SNPs and their interactions using least significance difference (LSD) method based on R package *agricolae* (version 1.3‐1).

## 3 RESULTS

### 3.1 Identification and genetic characteristic of genetic variants within *MXD3* gene

**Figure 1:**
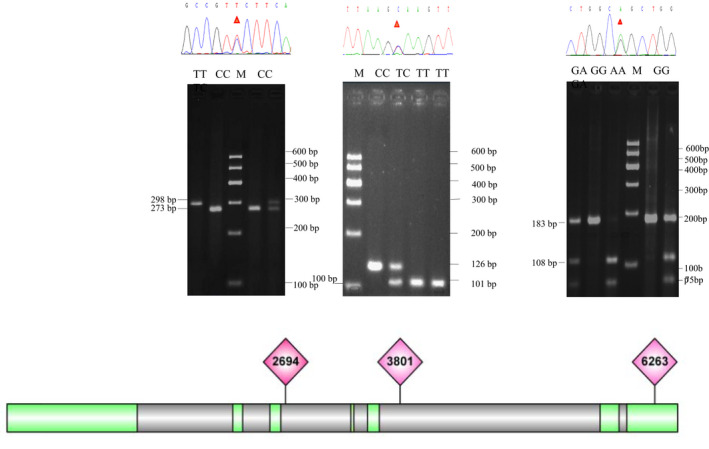
Electrophoresis patterns of three loci and structure diagram within *MXD3* gene. (a) g.2694 C>T: TT = 101+25 bp, TC = 126 + 101 + 25 bp, CC = 126 bp. (b) g.3801 T>C: TT = 298 bp, TC = 298 + 237 + 25 bp, CC = 237 bp. (c) g.6263G>A: GG = 183 bp, GA = 183 + 108 + 75 bp, AA = 108 + 75 bp. Some fragments were too short to be visible. M denote the size marker. Green colour represents the exons and the grey colour represents the introns of *MXD3*

The bovine *MXD3* is located at position ~40.1 Mb on Bos taurus autosome (BTA) 7 and consists of 6 introns and 7 exons. In this study, we identified three SNPs, i.e. g.2694 C>T (dbSNP Accession No. rs41593136, intron‐SNPs) in intron 3, g.3801 T>C (dbSNP Accession No. rs110929899, intron‐SNPs) in intron 5 and the g.6263 G>A (dbSNP Accession No. rs41255197, 3′UTR‐SNP), which was located in the 3′‐untranslated region (3′‐UTR) within the *MXD3* (Figure 1). In general, the predominant genotypes were heterozygous with frequencies above 0.500 except for the homozygous CC genotype at position g.2694 C>T, which was the most frequent in XN cattle. The frequencies of the C alleles of the two intron‐SNPs and the C and G allele in 3′UTR‐SNP were greater than 0.5 in both breeds. According to the results of chi‐squared test, the genotypes deviate from HWE except for g.3801 T>C in QC cattle and g.6263 G>A in both breeds (*p* > .05). In addition, *He* and *Ne* were approximately 0.5 and 2.0, respectively, and the *PIC* values were close to 0.37, indicating medium genetic diversity in the *MXD3* locus in the QC and XN populations (Table 1).

**Table 1:** Population genetic analysis of *MXD*3 in four Chinese native cattle breeds

Loci	Breeds	Genotype frequencies			AF		*P*(HWE)	*Ne*	*He*	*PIC*
		CC	TC	TT	C	T				
g.2694 C>T	QC(322)	0.320 (103)	0.559 (180)	0.121 (39)	0.599	0.401	*p* < .1	1.924	0.48	0.365
	XN(177)	0.458 (81)	0.384 (68)	0.158 (28)	0.650	0.350	*p* < .1	1.835	0.455	0.352
g.3801 T>C	QC(317)	0.320 (89)	0.559 (154)	0.121 (74)	0.524	0.476	*p* > .5^a^	1.996	0.499	0.374
	XN(177)	0.215 (38)	0.616 (109)	0.169 (30)	0.523	0.477	*p* < .1	1.996	0.499	0.374
		AA	AG	GG	A	G				
g.6263 A>G	QC(322)	0.211 (68)	0.509 (164)	0.280 (90)	0.466	0.534	*p* > .5^a^	1.991	0.498	0.374
	XN(177)	0.175 (31)	0.525 (93)	0.299 (53)	0.438	0.562	*p* > .5^a^	1.97	0.492	0.371

Abbreviations: AF, Allele frequencies; *He*, Expected heterozygosity; *Ne*, Effective allele numbers; *P*(HWE), *P* values of Hardy–Weinberg equilibrium; *PIC*, Polymorphism information content; QC, Qinchuan cattle; XN, Xianan cattle.

a Represented the breed was in Hardy–Weinberg equilibrium.

### 3.2 LD and haplotype information analysis

**Figure 2:**
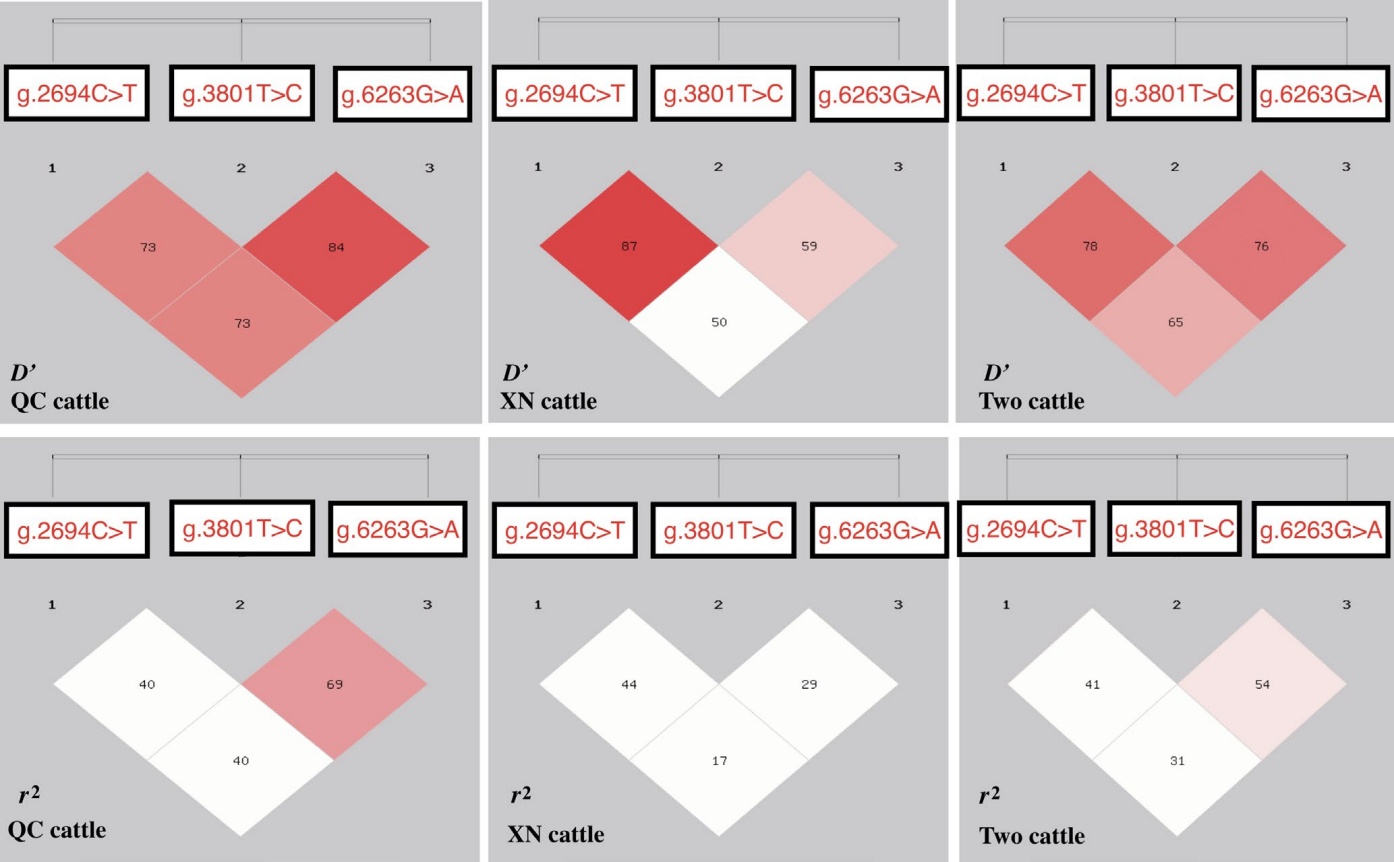
Linkage disequilibrium (LD) plots of *MXD3* gene in QC and XN cattle. (a) *D*′ only in QC cattle; (b) *D*′ only in XN cattle; (c) *D*′ in both QC and XN cattle; (d) *r* ^2^ only in QC cattle; (e) *r* ^2^ only in XN cattle; (f) *r* ^2^ in both QC and XN cattle. Colour scheme was on the basis of SHEsis *r* ^2^ scheme that is shown in percentage (%) and the *r* ^2^ value (%) between the pairwise loci were shown in each cell

Next, we calculated the LD parameters *r* ^2^ and *D*′ for all pairs of the three SNP loci (Figure 2). Moderate‐to‐strong LD was observed among the three loci in QC cattle (*D*′ ≥ 0.73 and *r* ^2^ ≥ 0.40), whereas in XN cattle strong LD only occurred between g.2694 C>T and g.3801 T>C loci ((*D*′ = 0.87 and *r* ^2^ = 0.44). Haplotypes often provide more reliable information than a single marker (Mokry et al., 2014), we determined the haplotypes of the three SNPs in the populations. Eight predicted haplotypes were found with the distributions of haplotypes in QC cattle and XN cattle shown in Table 2. The H1 haplotype (‐CCG‐) had the highest frequency in both breeds (about 44%), followed by H8 haplotype (‐TTA‐) with approximately 30%.

**Table 2:** The distribution of different haplotypes in three loci

Haplotype	Position of the three SNPs			QC	XN	Total
	g.2694T>C	g.3801T>C	g.6263A>G			
H1(CCG)	C	C	G	0.45	0.42	0.44
H2(CCA)	C	C	A	0.02	0.08	0.04
H3(CTA)	C	T	A	0.10	0.10	0.10
H4(CTG)	C	T	G	0.03	0.05	0.03
H5(TCA)	T	C	A	0.02	0.01	0.02
H6(TCG)	T	C	G	0.04	0.01	0.03
H7(TTG)	T	T	G	0.02	0.08	0.04
H8(TTA)	T	T	A	0.33	0.25	0.30

Abbreviation: SNP, single‐nucleotide polymorphisms.

### 3.3 Association analysis between single SNP and growth traits

An association analysis was performed to provide novel information for use in breeding programs of genetically improved cattle. The growth traits encompassing BH, BL, BW, ChC and HCH were significantly different in XN and QC cattle at 2 years of age (*p* < .001) based on an ANOVA analysis, and in general, the XN cattle showed better performance than QC cattle (Table 3). There was no significant association between the single SNP and the growth traits in the two cattle breed separately (Tables S3 and S4). Therefore, we analyzed the three variants in the two breeds combined and the results showed that the g.2694 C>T locus was significantly associated with all growth phenotypes except BH. Compared with CT and TT genotypes at g.2694 C>T locus, the contribution of CC genotype to BW values increased by 7.49% and 3.06%, respectively. A significant association was also observed between the g.3801 T>C locus and the five traits. Here, the TC genotype had 8.45% and 7.44% higher BW values than TT and CC genotypes, respectively. Although g.6263 G>A locus had no significant associations with growth traits, there was a tendency of the GA genotype to show somewhat better performance than other genotypes (Table 3).

**Table 3:** Association analysis of single SNPs with growth traits in the analysed sample

Marker	Locus	Genotype	Growth traits				
			BH (cm)	BL (cm)	BW (kg)	ChC (cm)	HCH (cm)
SNP1	g.2694 C>T	**CC(119)**	132.26 ± 6.46^a^	149.15 ± 17.06^a^	470.16 ± 122.10^a^	185.78 ± 12.85^a^	133.12 ± 8.27^a^
		CT(143)	132.01 ± 6.30^ab^	145.41 ± 15.61^b^	434.95 ± 119.07^b^	183.78 ± 13.42^ab^	131.00 ± 8.15^b^
		TT(49)	130.99 ± 4.90^b^	148.63 ± 12.56^a^	455.78 ± 97.92^a^	182.01 ± 11.14^b^	132.72 ± 6.25^a^
	*P*		.012	.01	<.001	.01	.00100
SNP2	g.3801 T>C	CC(76)	131.39 ± 6.45^ab^	145.32 ± 17.13^b^	433.74 ± 126.09^b^	182.24 ± 13.63^b^	131.26 ± 8.47^b^
		**TC(167)**	132.22 ± 5.95^a^	149.55 ± 14.80^a^	468.62 ± 115.50^a^	185.19 ± 12.94^a^	133.14 ± 7.44^a^
		TT(66)	130.45 ± 6.32^b^	144.12 ± 16.11^b^	429.01 ± 109.01^b^	181.74 ± 11.81^b^	130.35 ± 8.35^ab^
	*P*		.001	<.001	<.001	<.001	<.001
SNP3	g.6263 G>A	AA(60)	131.38 ± 6.49	146.38 ± 15.35^ab^	449.51 ± 118.46^ab^	183.80 ± 12.76^ab^	130.95 ± 8.36^b^
		**GA(153)**	132.11 ± 5.98	148.71 ± 15.10^a^	459.70 ± 115.07^a^	184.81 ± 12.69^a^	132.98 ± 7.40^a^
		GG(98)	131.06 ± 6.28	145.87 ± 17.12^b^	440.59 ± 122.13^b^	182.02 ± 13.38^b^	131.39 ± 8.48^b^
	*P*		.50	.58	.27	.13	.24
Breed		XN(141)	134.85 ± 4.29^a^	159.00 ± 6.24^a^	543.78 ± 5.54^a^	191.89 ± 8.36^a^	137.85 ± 3.37^a^
		QC(167)	127.73 ± 5.83^b^	133.65 ± 12.77^b^	339.62 ± 64.07^b^	173.73 ± 10.22^b^	125.06 ± 6.03^b^
	*P*	<.001	<.001	<.001	<.001	<.001	<.001

Note

LSD method was used for multiple comparisons in different genotypes at same phenotypes and values with different letters means different significantly, with *p* < .05 or 0.001 (a, b, c and d).

Abbreviations: BH, Body height (cm); BL, Body length (cm); BW, Body weight (kg); ChC, Ches circumference (cm); HCH, Hip cross height (cm); SNP, single‐nucleotide polymorphisms. The bold in the "Genotype" means the genotype showed higher pehnotype values.

### 3.4 Association analysis between combined SNPs and growth traits

To improve the reliability of the association results, we also conducted pairwise SNPs analyses (Table 4). The highest frequencies of different pairwise SNPs were CTTC (34.95%) at SNP1‐SNP2, and CTAG (25.94%) at SNP1‐SNP3 and TCAG (35.92%) at SNP2‐SNP3, respectively. Most of the pairwise variants showed impact on the different body traits (*p* < .01 or *p* < .05). For example, extremely significant associations were detected between SNP1‐SNP2 and BL, BW and HCH (*p* < .001). Also, significant associations were observed between SNP1‐SNP2 and the two growth traits, BH and ChC (*p* < .05). In addition, the CCTC combined genotype of SNP1‐SNP2 demonstrated higher performance than other combinations (Table 4). Furthermore, ten combined genotypes were identified when we conjoined the three SNPs. The data show a strong association between all growth traits and the various triple SNP combinations (Table 5). The growth traits value of cattle with CCCCGA combined genotype were 6.16% for BH, 17.55% for BL, 37.50% for BW, 8.56% for ChC and 10.61% for HCH higher than those of CTTTAA combined genotype (Table 5).

**Table 4:** Association analysis of pair‐wise SNPs with growth traits in the analysed sample

	CoG	Growth traits				
		BH (cm)	BL (cm)	BW (kg)	ChC (cm)	HCH (cm)
SNP1‐SNP2	CCCC(58)	131.52 ± 6.86^bc^	145.45 ± 18.71^bc^	442.67 ± 131.17^bc^	183.24 ± 13.91^b^	131.71 ± 8.97^b^
	CCTC(54)	134.00 ± 5.13^a^	155.09 ± 12.35^a^	513.80 ± 95.46^a^	189.42 ± 10.86^a^	136.01 ± 5.74^a^
	CCTT(6)	123.67 ± 5.65^d^	131.50 ± 17.12c	349.70 ± 81.73^c^	177.50 ± 10.45^b^	120.83 ± 6.08^d^
	CTCC(15)	130.87 ± 5.07^bcd^	145.60 ± 10.97^bc^	418.12 ± 110.07^bc^	179.73 ± 13.11^b^	130.23 ± 6.96^bc^
	CTTC(108)	131.44 ± 6.22^bc^	146.81 ± 15.44^bc^	447.04 ± 119.77^b^	183.01 ± 13.40^b^	131.79 ± 7.88^b^
	CTTT(19)	128.53 ± 7.32^cd^	137.26 ± 17.77^c^	379.52 ± 109.60^c^	178.11 ± 13.58^b^	127.05 ± 9.58^cd^
	TTCC(3)	131.33 ± 6.02^bcd^	141.33 ± 12.42^bc^	364.51 ± 67.83^c^	175.33 ± 10.21^b^	127.83 ± 4.80^bcd^
	TTTC(5)	129.80 ± 4.09^bcd^	148.80 ± 8.04^abc^	446.78 ± 92.08^bc^	186.60 ± 14.62^ab^	131.20 ± 5.80^bc^
	TTTT(41)	132.33 ± 4.96^ab^	149.15 ± 13.08^ab^	463.56 ± 98.68^b^	184.05 ± 10.76^b^	133.26 ± 6.32^ab^
	*P*	.001	<.001	<.001	.014	<.001
SNP1‐SNP3	CCAA(10)	133.90 ± 7.49^ab^	159.40 ± 9.29^a^	551.28 ± 90.71^a^	194.10 ± 11.64^a^	135.90 ± 8.81^ab^
	CCGA(53)	133.98 ± 5.84^a^	153.40 ± 14.25^ab^	499.87 ± 105.54^ab^	188.72 ± 10.93^ab^	135.73 ± 6.88^ab^
	CCGG(56)	130.22 ± 6.36^ab^	142.98 ± 18.56^d^	425.49 ± 126.29^c^	181.23 ± 13.37^c^	129.98 ± 8.47^d^
	CTAA(27)	130.70 ± 6.94^ab^	144.26 ± 17.07^cd^	441.17 ± 125.76^bc^	183.00 ± 12.93^bc^	129.81 ± 9.14^d^
	CTGA(83)	130.86 ± 6.09^ab^	144.82 ± 15.29^cd^	426.79 ± 116.01^c^	181.79 ± 13.47^c^	130.84 ± 7.49^bcd^
	CTGG(33)	131.64 ± 6.37^ab^	147.76 ± 15.20^bcd^	448.24 ± 122.06^bc^	181.55 ± 13.91^c^	132.38 ± 8.82^abcd^
	TTAA(23)	131.07 ± 5.42^ab^	143.22 ± 12.61^cd^	415.04 ± 97.66^c^	180.26 ± 11.02^c^	130.136.58^cd^
	TTGA(17)	132.18 ± 4.17^ab^	151.59 ± 11.83^abc^	484.04 ± 92.47^abc^	185.94 ± 10.62^abc^	134.21 ± 5.27^abc^
	TTGG(9)	134.11 ± 4.57^a^	156.89 ± 7.10 ^ab^	506.51 ± 69.77^ab^	188.67 ± 10.61^abc^	136.56 ± 4.42^a^
	*P*	.045	<.001	<.001	.003	<.001
SNP2‐SNP3	CCAA(7)	133.86 ± 4.63^ab^	155.29 ± 9.03^ab^	521.64 ± 95.11^a^	190.57 ± 8.70^ab^	134.43 ± 5.32^ab^
	CCGA(22)	134.36 ± 6.43^a^	155.77 ± 10.23^a^	521.29 ± 97.44^a^	189.91 ± 10.79^ab^	136.75 ± 7.48^a^
	CCGG(47)	129.63 ± 6.16^b^	138.94 ± 17.63^c^	381.28 ± 111.95^b^	177.40 + 13.32^c^	128.22 ± 7.89^c^
	TCAA(21)	133.71 ± 5.45^ab^	154.90 ± 11.51^ab^	521.43 ± 99.81^a^	190.95 + 11.56^a^	134.67 ± 8.01^ab^
	TCGA(111)	131.81 ± 5.96^ab^	147.69 ± 15.27^b^	448.87 ± 115.78^a^	183.99 ± 13.26^b^	132.43 ± 7.22^b^
	TCGG(35)	132.60 ± 6.17^ab^	152.23 ± 14.15^ab^	499.54 ± 109.20^a^	185.57 ± 12.04^ab^	134.49 ± 7.69^ab^
	TTAA(32)	129.30 ± 6.87^b^	138.84 ± 14.87^c^	386.53 ± 98.76^b^	177.63 ± 11.16^c^	127.75 ± 7.96^c^
	TTGA(18)	131.22 ± 5.16^ab^	146.22 ± 17.15^bc^	454.01 ± 109.61^a^	183.67 ± 10.03 ^bc^	131.78 ± 7.38^bc^
	TTGG(16)	131.88 ± 6.31^ab^	152.31 ± 14.07^ab^	485.87 ± 98.33^a^	187.81 ± 12.39^ab^	133.94 ± 8.90^ab^
	*P*	.02	<.001	<.001	<.001	<.001

Note

LSD method was used for multiple comparisons in different pairwise SNPs at the same phenotypes. The values with different letters means different significantly, with *p* < .05, .01 or .001 (a, b, c and d).

Abbreviations: BH, body height (cm); BL, body length (cm); BW, body weight (kg); ChC, Ches circumference (cm); HCH, hip cross height; SNP, single‐nucleotide polymorphisms.

**Table 5:** Association analysis of combined three SNPs with growth traits in the analysed sample

CoG	Growth traits				
SNP1SNP2SNP3	BH (cm)	BL (cm)	BW (kg)	ChC (cm)	HCH (cm)
CCCCGA(17)	135.76 ± 5.45^a^	158.65 ± 5.98^a^	545.53 ± 64.90^a^	191.71 ± 6.94^a^	139.06 ± 4.89^a^
CCCCGG(37)	129.23 ± 6.74^cd^	137.76 ± 19.19^de^	380.76 ± 118.54^c^	178.05 ± 14.18^c^	127.78 ± 8.47^de^
CCTCGA(32)	134.16 ± 5.26^a^	154.22 ± 12.94^a^	499.02 ± 101.69^a^	189.13 ± 11.48^a^	135.70 ± 5.77^ab^
CCTCGG(17)	132.82 ± 5.03^ab^	154.41 ± 12.29^a^	524.33 ± 88.53^a^	187.59 ± 9.10^ab^	135.41 ± 6.21^ab^
CTTCAA(15)	132.93 ± 5.64^ab^	152.73 12.13^ab^	506.48 ± 110.48^a^	188.13 ± 11.37^ab^	133.40 ± 8.65^bc^
CTTCGA(75)	130.90 ± 6.08^bcd^	144.83 15.69	428.17 ± 116.78^b^	181.83 ± 13.47^bc^	131.04 ± 7.45^c^
CTTCGG(18)	132.39 ± 7.22^abc^	150.17 ± 15.77^abc^	476.14 ± 123.61^ab^	183.67 ± 14.29^abc^	133.61 ± 8.96^bc^
CTTTAA(10)	127.40 ± 7.90^d^	130.80 ± 16.45^e^	340.94 ± 84.09^c^	175.30 ± 13.04^c^	124.30 ± 7.76^e^
TTTTAA(21)	130.88 ± 5.51^bcd^	142.76 ± 13.12^cd^	410.56 ± 100.85^bc^	179.00 ± 10.49^c^	130.14 ± 6.84^cd^
TTTTGA(12)	133.50 ± 3.32^ab^	154.42 ± 10.71^a^	515.51 ± 67.25^a^	188.33 ± 8.27^ab^	135.7 ± 53.96^ab^
*P*	.001	<.001	<.001	<.001	<.001

Note

LSD method was used for multiple comparisons in different combined SNPs at the same phenotypes. The values with different letters means different significantly, with *p* < .05, .01 or .001 (a, b, c, d and e).

Abbreviations: BH, body height (cm); BL, body length (cm); BW, body weight (kg); ChC, ches circumference (cm); HCH, hip cross height; SNP, single‐nucleotide polymorphisms.

### 3.5 Detection of expression levels of *MXD3* in seven different tissues

**Figure 3:**
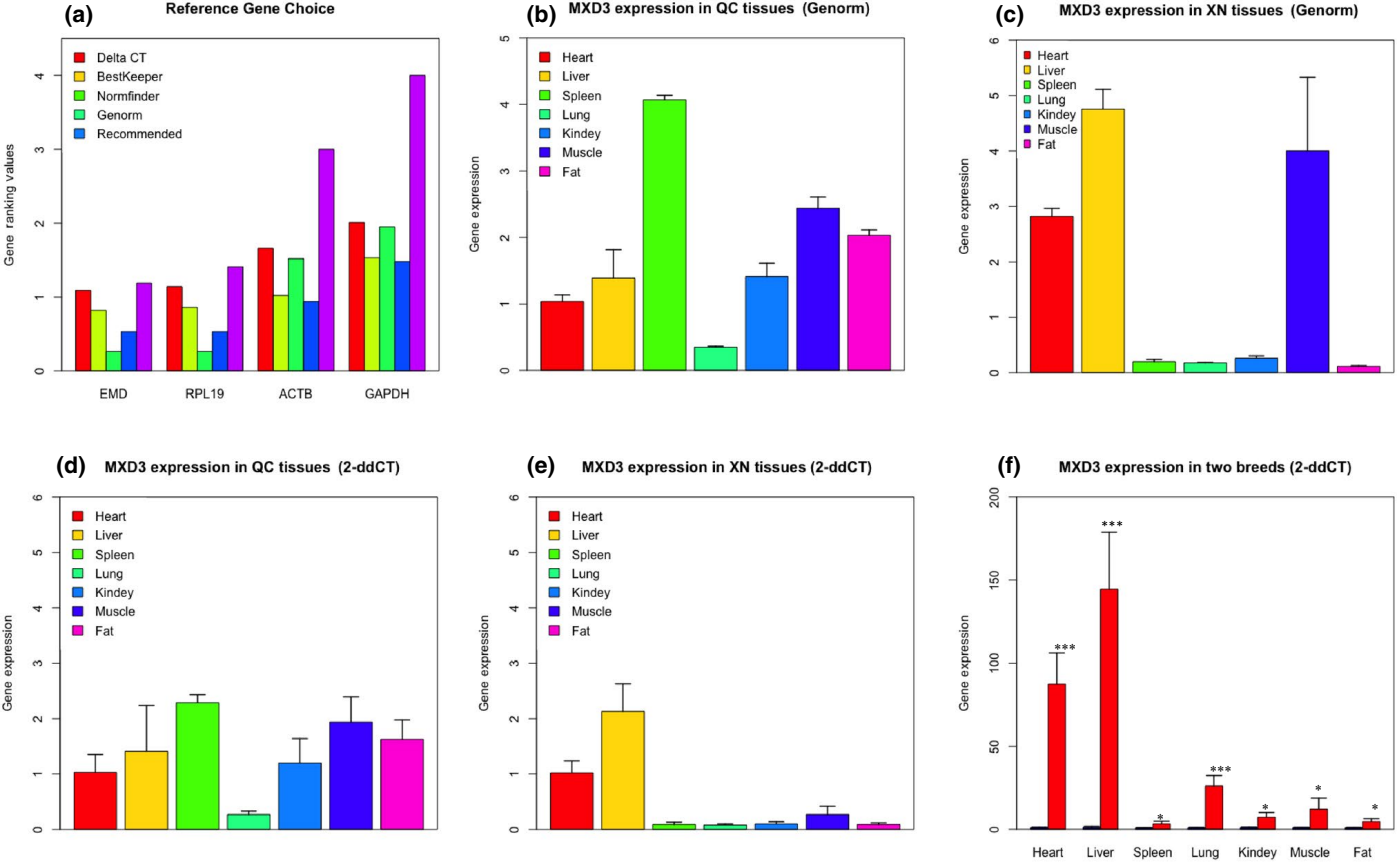
Expression profiles of *MXD3*. (a) Reference genes choice; (b) *MXD3* expression level in QC cattle using GeNorm methods. (c) *MXD3* expression level in XN cattle using GeNorm methods; (d) *MXD3* expression level in QC cattle using 2^−ΔΔ^ *^C^* ^T^ methods. (e) *MXD3* expression level in XN cattle using 2^−ΔΔ^ *^C^* ^T^ methods. (f) *MXD3* expression level in two breeds using 2^−ΔΔ^ *^C^* ^T^ methods. ***denote *p* < .001 and *denote *p* < .05. “I” represented standard deviations of three biological replicates for each tissue

To assess and compare the expression level of *MXD3* in different tissues, we first determine which reference genes would be most suitable for normalization. As shown in Figure 3a, geNorm ranked *RPL19* and *EMD* as the best combination for gene expression normalization in various cattle tissues. Delta CT, BestKeeper and Normfinder suggested a single reference gene *EMD* (Figure 3a and Figure S3). Therefore, we used two approaches to normalize the *MXD3* expression level based on our results: (a) A geometric mean of the two most stably expressed genes (*RPL19* and *EMD)* combined as suggested by Vandesompele et al. (2002), (b) Using a single best gene *EMD* as suggested by the 2^−ΔΔ^ *^C^* ^T^ method. The data shown in Figure 3 demonstrate that the *MXD3* expression levels as measured by the two different methods were highly consistent. Furthermore, we observed that the *MXD3* gene showed the highest expression level in spleen, followed by muscle and fat in QC cattle, whereas, in XN cattle, the highest expression was found in the liver followed by heart and muscle (Figure 3b–e). It is also noteworthy that the *MXD3* was differently expressed in the seven tissues between two breeds based on *t* test (*p* < .001 or *p* < .05) (Figure 3f).

## 4 DISCUSSION

Cattle have the ability to convert low‐quality forage into high‐quality food such as milk and beef, which are important sources of human nutrition. However, Chinese indigenous cattle with inferior productivity indexes cannot satisfy the increasing demand for beef production in China. Over recent years, researchers have investigated the genetic basis of many complex phenotypes and many polymorphisms in various functional genes have been reported associated with cattle economic important traits, suggesting they may be useful as markers for the genetic improvement of cattle (Bouwman et al., 2018). In this study, we investigated the expression profiles of the *MXD3* gene and evaluated the possible association of sequence variants with growth traits in XN and QC cattle. The *MXD3* plays important regulatory roles in physiological processes such as cellular proliferation and differentiation and mutations in *MXD3* are associated with tumorigenesis, i.e. leukaemia and medulloblastomas (Barisone et al., 2012; Ngo, Barisone, Lam, & Daz, 2014; Ngo et al., 2019; Satake et al., 2014, 2016). In our study, we identified three SNPs within *MXD3* (SNP1: g.2694 C>T; SNP2: g.3801 T>C and SNP3: g.6263 G>A) and excavated the potential relations between three SNPs and cattle growth traits. Our data showed ubiquitous expression of *MXD3*, yet at highly variable levels, in all the tissues that we investigated. Importantly, the expression data were obtained using two different approaches for accurate normalization. Thus, many commonly used housekeeping (HK) genes employed in qRT‐PCR studies often exhibit tissue‐dependent variation (Lin & Redies, 2012). Therefore, we first carefully determined the optimal strategies for normalization using Delta CT, BestKeeper and Normfinder methodology in the RefFinder tool, resulting in reliable and consistent expression data. In QC cattle, *MXD3* was highly expressed in spleen, muscle and fat tissues, while transcription was highest in liver, muscle and heart tissues in XN cattle. Overall, this is in accordance with the finding that the *MXD3* have an important role in regulating B‐cell differentiation (Gore, Lantner, Hart, & Shachar, 2010) and furthermore that the *MXD3* was highly expressed in obese individuals (Shimada et al., 2014). It is noteworthy that the *MXD3* expression profiles were quite different between the two genetically different cattle breeds. Thus, the QC cattle is one of the top five Chinese yellow indigenous cattle breeds, whereas the XN cattle is a new crossbreed with French Charolais cattle and Chinese Nanyang cattle. It is possible that the observed breed‐specific *MXD3* expression profiles may contribute to the phenotypic differences between QC and XN cattle.

We identified two intronic SNPs (g.2694 C>T and g.3801 T>C) and one SNP (g.6263 G>A) in the 3′UTR of *MXD3*, which we suggest could be used to promote the genetic improvement of Chinese cattle. Thus, a major result of this study is the observations of strong associations between pairwise and triple SNP combinations and the performance traits measured in the two breeds. Another noticeable example of non‐coding SNPs with phenotypic impact is a mutation 4251 nt (C>T) in intron 1 of the growth hormone‐releasing hormone (GHRH) gene, which was significantly associated with body weight in QC cattle (Zhang et al., 2012). Non‐coding SNPs may have effects on mRNA metabolism by transcriptional enhancement or repression, or by influencing nucleosome‐positioning elements in the gene or via an effect on the assembly of spliceosome components (Le Hir, Nott, & Moore, 2003). Interestingly, the TargetScan algorithm predicts that the 3′UTR‐SNP (g.6263 G>A) in *MXD3* may decrease its interaction with bta‐miR‐22‐3p. This agrees well with the previous observation that bta‐miR‐22‐3p is associated with cattle development based on genome‐wide profiles on muscle tissue between fetal and adult QC cattle (Agarwal, Bell, Nam, & Bartel, 2015; Huang et al., 2014; Sun et al., 2015). However, further studies are needed to confirm the interaction between miR‐22‐3p and 3′‐SNP in *MXD3* and to better understand how this affects complex phenotypes.

The detection of gene variants associated with economically important traits has provided insights into the genetic architecture of complex traits and diseases in cattle (Bouwman et al., 2018; Suravajhala, Kogelman, & Kadarmideen, 2016). Recently, researchers have paid increased attention to the integration of muti‐omic data, which will provide a system‐level understanding of the biology of complex traits and be instrumental in improving sustainable breeding of productive and healthy animals (Suravajhala et al., 2016). Thus, future use of present and emerging omics‐technologies will be applied to collaboration with breeders and farmers.

## CONFLICT OF INTEREST

The authors declare that they have no conflict of interest.

## AUTHOR CONTRIBUTION

DH performed the experiments, analysed the data, and DH and BT drafted the manuscript. JSB and XW improved the manuscript. SJP, XYL and YZH developed and collected the samples. HC and XW conceived and designed the study, reviewed the study and supervised the research.

## ETHICAL APPROVAL

All animal procedures were carried out according to protocols approved by the College of Animal Science and Technology, Northwest A&F University, China.

## Data Availability

The data that support the findings of this study are available from the corresponding author upon reasonable request.
