# Supplementary material for: Expression profiles of the MXD3 gene and association of sequence variants with growth traits in Xianan and Qinchuan cattle
Source: Vet Med Sci. 2020 Mar 5;6(3):399–409. doi: 10.1002/vms3.251 (PMC7397896; doi:10.1002/vms3.251)
Supplement: Supplementary file 1 [file VMS3-6-399-s001.docx]

**Additional files**

**Table S1**. Primers for detecting the SNPs of *MXD3* gene and the primers for RT- qPCR

| Primers for detection SNPs | | |  | Primers for RT-qPCR | | |
| --- | --- | --- | --- | --- | --- | --- |
| Primers | AL(bp)^a^ | AT(℃)^b^ | Sequences of primers | Genes | AL(bp) | Primer pairs sequences (5’–3’) |
|  |  |  |  |  |  |  |
| P1 | 697 | 55 | F:TGGACCTGTCACAGTGTATC | *MXD3* | 127 | F- GGTTTCGCAGGGCTTCC |
|  |  |  | R:CCTCTTTCCTGTAAGTTCGG |  |  | R- AGACTTGGATGTTGCTGGC |
| P2 | 499 | 56 | F:GACTATTCCCATTCGCCACAG | *GAPDH* | 145 | F-TGAGGACCAGGTTGTCTCCTGCG |
|  |  |  | R:ATCATTTGGTGTTTCTGGGC |  |  | R-CACCACCCTGTTGCTGTAGCCA |
| P3 | 671 | 61 | F:TTGTTGCCCCGTGTCAGTT | *LRP10* | 139 | F- CCAGAGGATGAGGACGATGT |
|  |  |  | R:ACGACAGGAGTCCGTTTGC |  |  | R-ATAGGGTTGCTGTCCCTGTG |
| P4 | 607 | 58 | F:TTTGGGTGACTTGTGTGGCT | *EMD* | 100 | F- GCCCTCAGCTTCACTCTCAGA |
|  |  |  | R:AGTGTCTTACCTGGATGTGC |  |  | R- GAGGCGTTCCCGATCCTT |
| P5 | 732 | 63 | F:AGACTGGAGAGGGCTGTTTG | *PPIB* | 143 | F- ACACCAACGGCTCCCAGT |
|  |  |  | R:AGGTTTTCAGCAGGGGACAG |  |  | R- AGGCTTGTCCCGACCATC |
| P6 | 762 | 51 | F:TTGCCTCTGGTCTCATCTTG | *ACTB* | 105 | F- CTTCCAGCCGTCCTTCCT |
|  |  |  | R:CAAGTGATGTGAAGGAGTGG |  |  | R- TGTTGGCATACAGGTCCTTTC |
| P7 | 814 | 60 | F:CCCAACCATCCAGACAGAAC | *RPL19* | 119 | F-GGGTACTGCCAATGCTCGAA |
|  |  |  | R:CTGGGCCAAGTGCTATAAAC |  |  | R-TGTGATACATGTGGCGGTCA |

**Note**: AL ^a^: Amplifed length; AT ^b^, Annealing temperature].

Table S2. Identification of genetic variants in *MXD3* gene

| SNPs | Primer sequence | Tm | FPR | PCR-RFLP pattern |
| --- | --- | --- | --- | --- |
| g.2964T>C | F:5'-TCCAGTCCACAGGAGGAGGAAGCGG-3'  R:5'-TGGGCAGTGGAGAGACCCGATG**TC**G-3' | 60℃ | *Taq*Ⅰ | 126/101+25 bp |
| g.3800T>C | F:5'-AAGAGGATTGTTCTGAGGCTTT**GC**G-3'  R: 5'-CTCTCAACCCTTTTCCCCCTTCTGG-3' | 55℃ | *Hha*Ⅰ | 298/273+25 bp |
| g.6263A>G | F:5'-CTTGGAGTCACTGTTGGAAT-3'  R:5'-GTTTAGGAAGGAACATGAGG-3' | 60.5℃ | *Pvu*Ⅱ | 183/108+75 bp |

**Note**: The underlined bases showed mismatches to create restriction sites. FPR: restriction enzyme used for forced PCR-RFLP

Table S3. Association analysis of single SNPs with growth traits in QC cattle

| Marker | Locus | Genotype | Growth traits | | | | |
| --- | --- | --- | --- | --- | --- | --- | --- |
|  |  |  | BH (cm) | BL (cm) | BW (kg) | ChC (cm) | HCH (cm) |
| SNP1 | g.2694 C>T | CC(42) | 126.70±5.60 | 130.12±13.74 | 329.50±64.87 | 172.90±9.64 | 124.04±6.09 |
|  |  | CT(78) | 127.64±5.98 | 134.32±11.68 | 340.51±61.82 | 173.55±10.32 | 125.11±6.13 |
|  |  | TT(21) | 130.12±5.36 | 138.24±11.34 | 362.35±63.07 | 176.62±10.80 | 127.21±5.08 |
|  | *P* |  | 0.100 | 0.080 | 0.155 | 0.376 | 0.145 |
| SNP2 | g.3801 T>C | CC(39) | 127.44±5.31 | 133.33±11.98 | 333.56±56.89 | 172.61±8.71 | 124.84±5.35 |
|  |  | TC(63) | 127.95±5.68 | 134.41±12.03 | 341.72±65.23 | 173.73±10.91 | 125.45±5.89 |
|  |  | TT(36) | 127.65±6.79 | 133.53±13.97 | 346.13±68.86 | 175.33±10.73 | 124.66±7.03 |
|  | *P* |  | 0.91 | 0.89 | 0.68 | 0.51 | 0.79 |
| SNP3 | g.6263 G>A | AA(32) | 128.29±6.94 | 135.59±12.71 | 355.26±68.48 | 176.31±10.99 | 125.15±7.18 |
|  |  | GA(64) | 128.26±5.48 | 134.64±12.06 | 343.42±60.43 | 174.19±9.91 | 125.96±5.35 |
|  |  | GG(44) | 126.64±5.43 | 131.65±12.64 | 325.70±61.84 | 171.45±9.71 | 123.82±5.94 |
|  | *P* |  | 0.50 | 0.32 | 0.11 | 0.11 | 0.24 |

Note: BH: Body height (cm); BL: Body length (cm); BW: Body weight (kg); ChC: Ches circumference (cm); HCH: Hip cross height (cm).

| Marker | Locus | Genotype | Growth traits | | | | | |
| --- | --- | --- | --- | --- | --- | --- | --- | --- |
|  |  |  | BH (cm) | BL (cm) | BW (kg) | ChC (cm) | HCH (cm) |  |
| SNP1 | g.2694 C>T | CC(77) | 135.20±4.70 | 159.29±5.76 | 547.46±57.79 | 192.76±7.91 | 137.78±3.29 |  |
|  |  | CT(65) | 135.04±3.77 | 158.67±6.66 | 547.20±55.20 | 192.06±8.93 | 138.07±2.79 |  |
|  |  | TT(28) | 133.42±4.06 | 156.42±6.19 | 525.85±47.18 | 189.14±8.01 | 136.85±3.00 |  |
|  | *P* |  | 0.15 | 0.112 | 0.174 | 0.145 | 0.213 |  |
| SNP2 | g.3801 T>C | CC(35) | 135.42±3.33 | 159.48±5.58 | 551.82±60.08 | 193.28±8.78 | 138.08±2.76 |  |
|  |  | TC(104) | 134.79±4.43 | 158.72±6.38 | 545.4±55.89 | 192.13±8.32 | 137.79±3.21 |  |
|  |  | TT(30) | 133.80±3.55 | 156.83±6.28 | 528.46±46.36 | 189.43±7.85 | 137.16±2.92 |  |
|  | *P* |  | 0.27 | 0.20 | 0.21 | 0.16 | 0.47 |  |
| SNP3 | g.6263 G>A | AA(28) | 134.89±3.54 | 158.71±5.78 | 557.21±52.15 | 192.35±8.63 | 137.57±2.83 |  |
|  |  | GA(88) | 134.63±4.30 | 158.45±6.81 | 541.55±60.41 | 192.17±8.47 | 137.69±3.16 |  |
|  |  | GG(53) | 134.86±4.08 | 158.60±5.50 | 540.37±47.91 | 191.18±8.21 | 137.92±3.09 |  |
|  | *P* |  | 0.93 | 0.979 | 0.117 | 0.759 | 0.864 |  |

Table S4. Association analysis of single SNPs with growth traits in XN cattle

Note: BH: Body height (cm); BL: Body length (cm); BW: Body weight (kg); ChC: Ches circumference (cm); HCH: Hip cross height (cm).

**
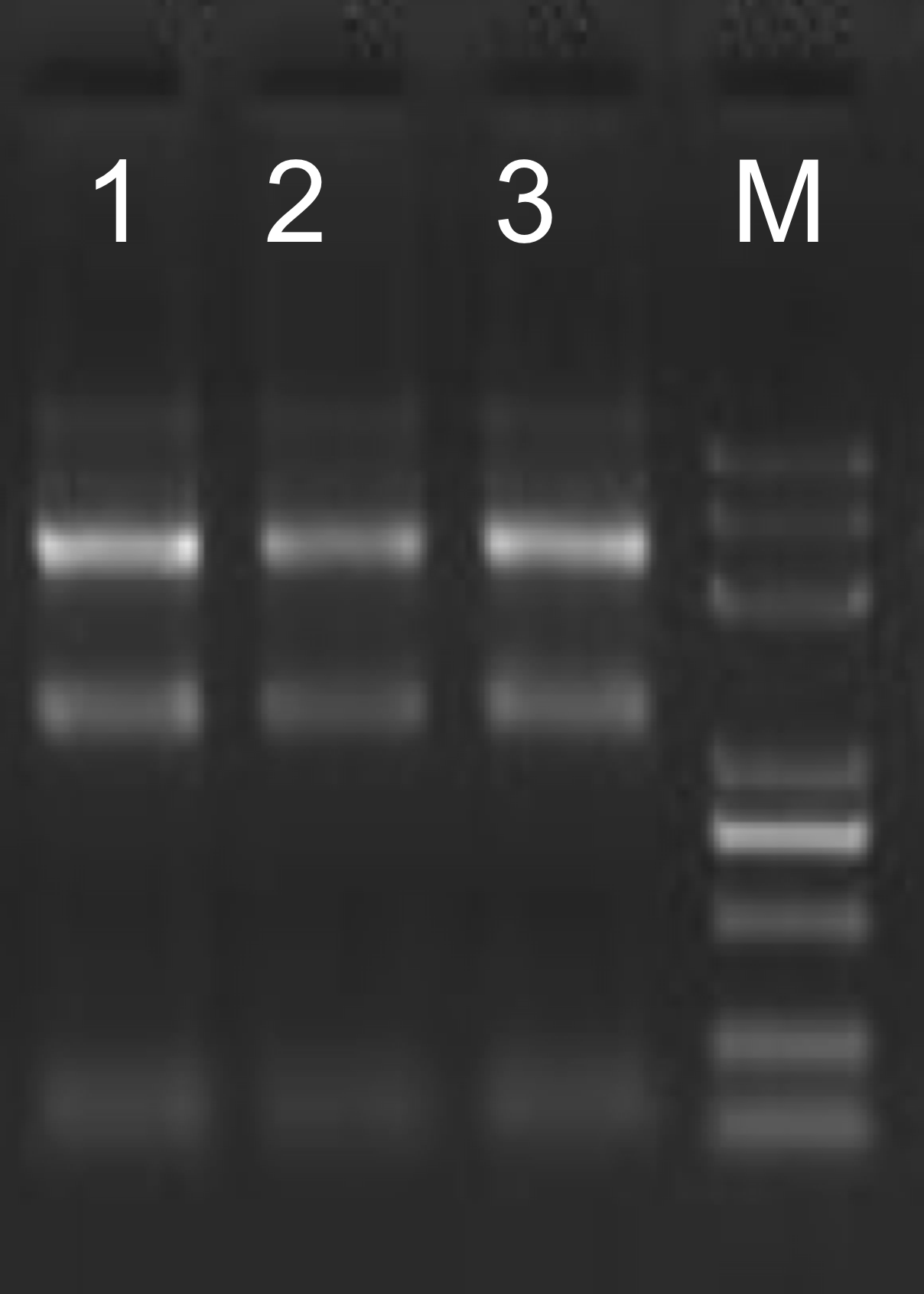
**

Figure S1. The RNA quality identified by 0.8% agarose gel electrophoresis.

**
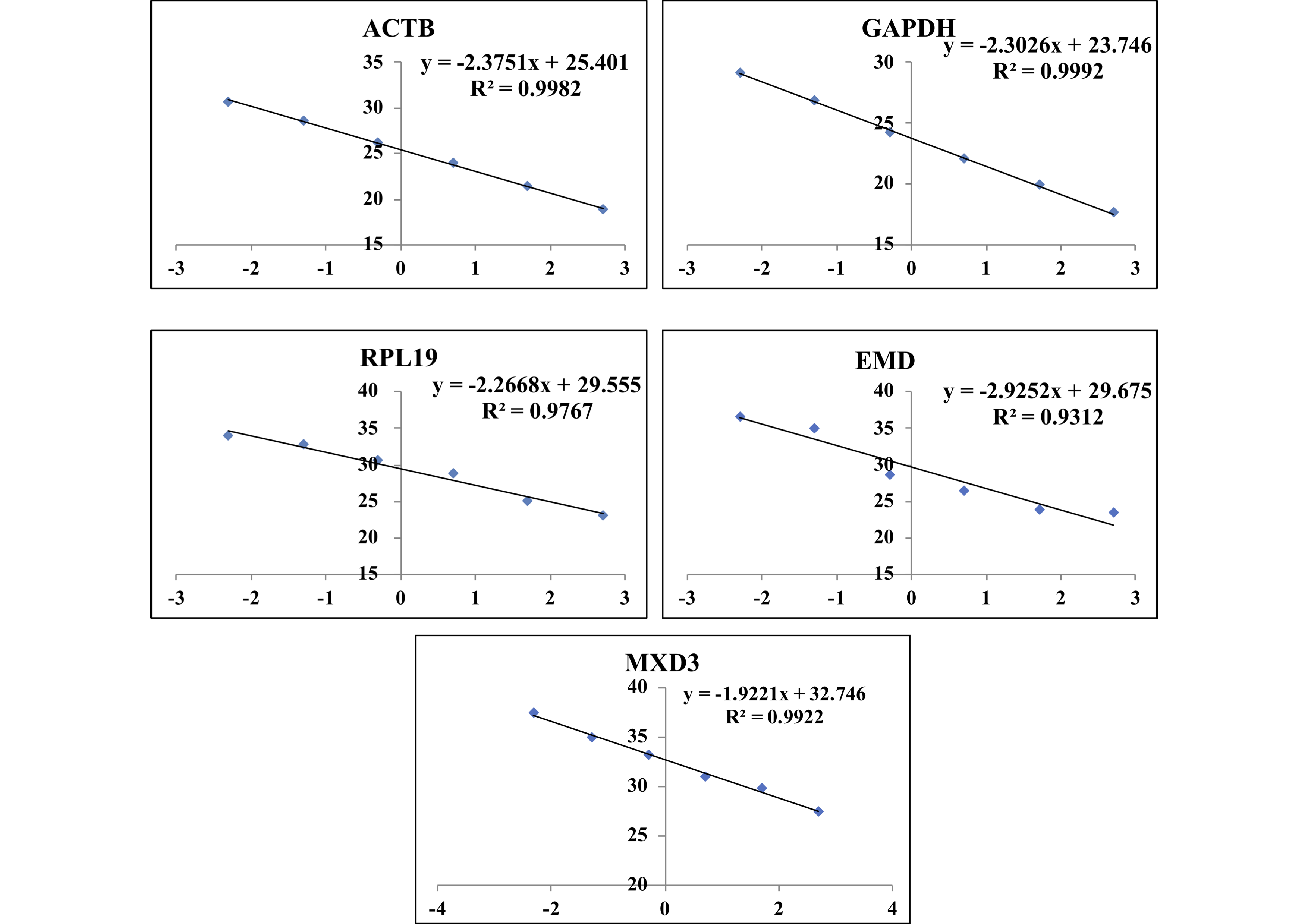
**

Figure S2. The primers amplification efficiency.

**
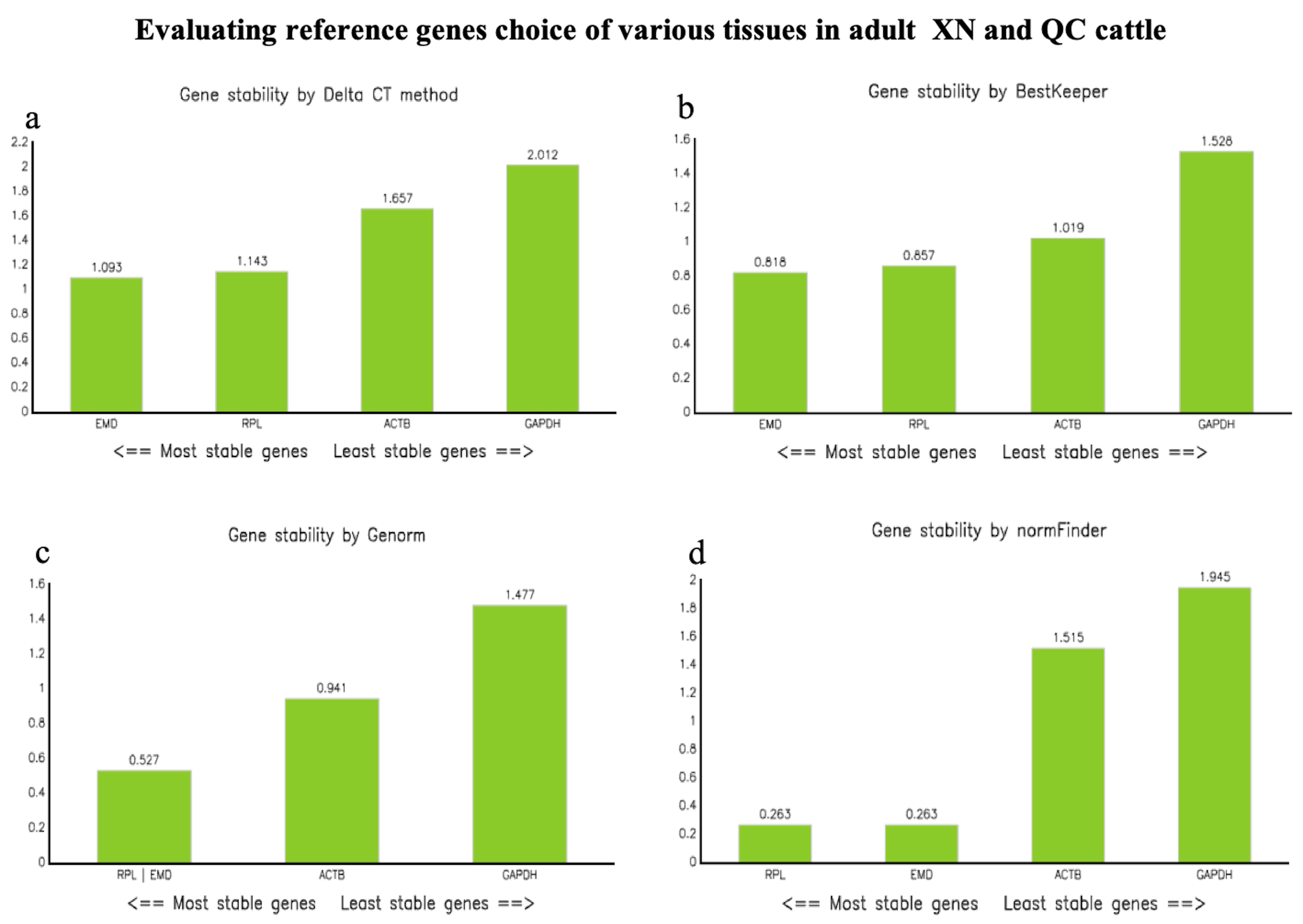
**

**Figure S3** Evaluating reference genes of various tissues in adult XN and QC cattle.(a)Delta CT method, (b) BestKeeper, (c)Genorm, (d) normFinder. The lower value the reference gene more stably.
